# Medical Opinion and Movement

**Published:** 1910-04-16

**Authors:** 


### Medical Opinion and Movement.

### Foreign Body Three Years in a Bronchus.

Dr. Sirotinine, of St. Petersburg, records the following interesting case. A man, aged 50, was admitted to hospital suffering from acute pleuro-pneumonia of the right side. Soon after admission he was suddenly seized with a severe pain under the third right rib, followed by an attack of spasmodic coughing during which he expectorated a fish-bone of a sturgeon. Soon afterwards another coughing attack resulted in the expulsion of a sort of membrane, which probably formed a capsule enclosing the foreign body in the bronchus. It was then elicited from the patient that the fish-bone had been swallowed three years before, and that at the time he had a severe attack of coughing which lasted about three hours and was accompanied by acute pain under the third rib to the right of the sternum. The cough continued and the patient consulted a number of doctors, but they refused to believe that the bone had remained in the chest. For over a year he was treated for bronchitis and emphysema. Then he had hæmoptysis. The pain in the chest diminished gradually and eventually only occurred on a severe attack of coughing, but he was unable to lie on the right side without exciting an attack of coughing. Bronchoscopy showed at a distance of 32 centimetres from the angle of the mouth the lumen of the bronchus filled to the extent of one-third by granulation tissue. This point would correspond to the level at which the right bronchus divides into three branches. The pulmonary and pleuritic inflammation rapidly subsided and the patient made a complete recovery.

### Adrenalin an Antidote to Strychnine.

Drs. W. Falta and L. Jvcovic report in the *Berliner Klinische Wochenschrift* a series of research experiments carried out by them to determine the antagonism between the actions of various cardiac poisons, and they find that, experimenting on the frog's heart, adrenalin is capable of annulling the effect of strychnine. The heart was exposed and three or four drops of a 2 per cent. solution of strychnine nitrate were allowed to fall upon it. At the end of about 30 seconds the heart was thrown into tetanus on percussion, the cardiac contractions becoming slower and slower, and finally ceasing with the heart in diastole. If now three or four drops of 1 in 1,000 adrenalin solution are placed on the heart, cardiac contractions soon recommence and gradually increase in rapidity till the heart beats more rapidly than normal, and if kept moistened with sodium chloride solution it may continue to beat for half an hour, eventually stopping in systole. Moreover, the tetanic spasm can no longer be produced by percussion. Exner had already shown some time ago that considerably larger doses of strychnine can be injected intraperitoneally in guinea-pigs and rabbits if preceded by injections of adrenalin, but this author explained the fact on the ground that the contraction of the blood-vessels produced by the adrenalin retarded the absorption of the strychnine. The present authors, however, are of opinion that the adrenalin acts as a direct antidote to strychnine, and they find that after injections in guinea-pigs absorption of the strychnine takes place equally quickly with or without the adrenalin, and that the same neutralising effect is produced when the two solutions are injected into different parts of the body.

### Inhalations of Ether an Antidote to Cocaine.

Another antidote of perhaps more practical value consists in inhalations of ether for cocaine-poisoning. Injections of ether are a common practice to combat cardiac failure in cocaine-poisoning, but Dr. Engstad, of Grand Forks, U.S., recommends that the ether be inhaled. He appears to have had experience of several cases of accidental poisoning arising from cocaine anæsthesia. At first he had recourse to injections of strychnine and morphine, but he found their action was too slow to be depended upon where life was really in danger. Ether inhalations, on the other hand, rapidly stimulate the respiratory and vasomotor centres, and at the same time exercise a powerful tonic effect upon the cardiac muscle. In all these respects, therefore, ether acts as a direct antagonist to cocaine. Under the influence of the inhalations the pulse becomes fuller and recovers its tone, mental excitement abates, and the other symptoms of intoxication rapidly disappear. The etherisation should only be pushed to a slight degree of narcosis, and for this purpose the ether should be administered by the drop method on an open mask, so as not to hinder a free access of air to the lungs. The author claims to have succeeded by this method in the recovery of patients almost despaired of. He suggests that the same method would be applicable in cases of stovaine-poisoning.

### Varicose Veins.

Apart from the ordinary surgical treatment, a number of methods of treatment of varicose veins have been devised consisting of injections of various substances, such as alcohol, ergotine, perchloride of iron, etc., with the object of producing either a coagulation of the blood in the vessels or an inflammation of the vessel walls. None of these injections, however, are free from danger, and have consequently fallen into disuse. Dr. Scharff, of Stettin, now reports successful results from the injection into the veins of a weak solution of perchloride of mercury. He finds by experiment that varicose veins are affected by such injections differently from healthy veins—that, for instance, 1 c.c. of a 1 per cent. solution produces hardly any local reaction in a healthy vein, but in an affected vein excites inflammation with thrombosis and even gangrene of the adjoining tissues. For purposes of injection into the various veins he uses strengths of 1 in 3,000 and 1 in 5,000 of perchloride of mercury in normal saline, and commences with injections of 0.5 c.c. of the weaker solution, and gradually increases the dose to 5 c.c., and, if necessary, passes on to the stronger solution. Superficial veins of small calibre require smaller doses of the perchloride. It is necessary, however, that the solution comes into contact with the whole of the endothelium of the varices. For this purpose he advises that the elastic bandage applied to the limb to make the veins obvious should not be removed till after the injection is completed. Several injections may be carried out at different points at one sitting. The necessary interval between the sittings depends upon the reaction. The injection should be almost painless. If an intense burning sensation is produced, it is due to the needle penetrating the wall of the vessel and entering the surrounding tissue. The author claims to have carried out the treatment in 90 cases, and to have obtained satisfactory and lasting results.

### The Removal of Plaster Appliances.

A simple and very practical method of rapidly removing splints, bandages, and other apparatus made of plaster of Paris is described by Max Stransky in the *Medizinische Klinik.* A common pocket-knife and a little vinegar are all the appliances necessary. A cotton-wool swab is soaked in the latter, which is then applied to the plaster surface along the line where it is proposed to divide the apparatus. After waiting a couple of minutes the surgeon is able, without the slightest difficulty and without causing the patient any pain, to divide the apparatus along the line softened by the vinegar. The author has employed his method for the past twenty years with the greatest success. He has been able by it to remove in a minute and a half a plaster thigh-bandage consisting of 80 turns.

### "Pleuresies Bloquees."

In a recent number of *La Presse Médicale* Mosny and Stern, under the heading "Pleurésies Bloquées," discuss those forms of pleurisy with effusion which, while giving all the physical signs of the presence of fluid, do not yield it on aspiration. It is not long ago that Dufour drew attention to the existence of chronic effusions of this nature, and since the publication of his observations cases have been recorded of acute pleurisies with effusion occurring which could not be aspirated. The authors of the present study divide "pleurésies bloquées" into two varieties, namely, complete and partial. In the first, although the diagnosis from the physical signs is positive as to the presence of fluid, the effects of aspiration are quite negative, and the true nature of the condition is often only made manifest at the autopsy. In the second variety a few cubic centimetres of fluid may be drawn off, but then of a sudden all efforts at further evacuation are negative, despite the certainty that more fluid is present. Partial "blockage" may also occur in some cases of bilateral effusion, it being possible to evacuate a large quantity of fluid from one side, whereas only a small quantity can be drawn off immediately afterwards from an obviously large accumulation on the opposite side. The authors believe that this is caused by the immobilisation of the mediastinum which is drawn over to the side first emptied and so cannot fill up the void on the opposite side, with the result that the fluid remains in the pleural cavity. In such cases it is easy to evacuate this effusion on the following day when the mobility and elasticity of the parts have returned. In acute pleurisy two conditions are necessary to effect this "blockage" of the fluid: (1) Pulmonary splenisation, which deprives the lung of its elasticity, renders it immobile, and prevents it filling up the vacuum left by the abstraction of fluid; and (2) the existence of basal adhesions which also immobilise the diaphragm and isolate the effusion by a zone of pleural adhesions. In chronic pleurisy a more or less complete envelope is formed round the fluid by the greatly thickened pleura, the sclerosed and often retracted thoracic parietes, the sclerosed and inelastic lung and the diaphragm immobilised by rigid pleural adhesions. It is obvious that fluid contained in such a cavity cannot be drawn off in any quantity by simple aspiration. It is possible to remove such collections of fluid by replacing the liquid by gas. The authors recommend the use of sterilised air, oxygen being too quickly and nitrogen too slowly absorbed to be used pure. They advise that at most an equal volume of air should replace the fluid, it being often better to employ only half the volume. As soon as the fluid ceases to flow on aspiration, or the patient complains of thoracic pain and oppression, a little air should be admitted. A further quantity of fluid can then be removed and more air admitted if necessary. As regards treatment the authors regard aspiration as useless in the case of acute pleurisy where the effusion is rich in polynuclear and endothelial cells, such as is often the case in tubercle, for the fluid will collect again immediately. In chronic tuberculous effusions, however rich in lymphocytes, evacuation is advantageous and should be performed at once. This should be repeated if the fluid collects again, for it is dangerous to leave such fluid in a sealed cavity owing to the risks of suppuration occurring, an event, however, which unfortunately cannot always be prevented by aspiration.

### Cerebral Tumours.

Some very important points connected with the treatment of brain tumours are dealt with by Mr. D. Armour in a paper reported in the *West London Medical Journal.* The author pleads for earlier diagnosis and operation, which are still much neglected and are necessary before operators can show results equal to those attained in other branches of surgery. He proceeds to emphasise the peculiar features of many syphilitic cerebral tumours, which may develop in patients who present no signs of other specific lesions, past or present; have often very slow onset and gradual development, yet are steadily progressive in spite of active anti-syphilitic treatment and may even appear during a mercurial course; may be of very hard fibrous consistency; and are curable only by surgical ablation. Instructive cases are quoted to illustrate these points, which are well worth the attention of practitioners. Of palliative operations the author has much to say. Decompression should be undertaken when radical cure is impossible in an early stage before vision begins to fail. A grave responsibility rests upon the medical attendant who allows a patient to become blind from optic atrophy due to tumour. To effect decompression effectually trephining is the only reliable means; puncture of the lateral ventricle or of the spinal theca cannot be compared with it, and the latter especially is not only unreliable but has distinct dangers of its own. In the operation the two important points to remember are: to open the skull over as silent and unimportant an area of the cortex as possible, and to prevent an unnecessarily large hernia cerebri by using muscles and fasciæ to act as a check upon it. Two-stage operations are as a rule the best, but cases must be decided upon their merits of urgency, shock, interference with respiratory and cardiac centres, etc.

---

### The Affected Side in Hemiplegia.

In the *Quarterly Journal of Medicine* Dr. Ernest Jones discusses in a most exhaustive paper the old belief that the side affected by a hemiplegia is of considerable value in the diagnosis of the nature of the affection. This opinion, which has been taught for more than a century, in spite of occasional protests to the contrary, has never referred to inflammatory lesions or tumours, which are accordingly excluded from consideration. But it is still generally held, he says, that a hemiplegia on the right side is presumptive evidence of either thrombosis or embolism, whereas one on the left similarly points to cerebral hæmorrhage or hysteria. The question would not be important, except as one of theoretical interest, were it not that the diagnosis of hysterical from organic hemiplegia and the differential diagnosis of the three arterial lesions themselves are often still very difficult. As material upon which to work for the purposes of this research the author has analysed 154 unpublished cases of cerebral arterial lesions from the autopsy records of University College Hospital; 626 clinical cases of hemiplegia personally observed during six years, mostly in London infirmaries; 373 cases of hysterical hemiplegia from the literature; 431 cases of organic hemiplegia similarly collected; and 3,697 cases of organic hemiplegia from published post-mortem records. Out of this total of 5,281 cases, in 3,539 the nature of the lesion was definitely determined by post-mortem examination. The conclusion drawn from the very critical study to which Dr. Jones has subjected the series is that there is no evidence to indicate that either the lesion or the hemiplegia is more apt to affect one side than the other; the general teaching to the contrary is not founded upon evidence. If such a belief does really exist to the extent which the author believes, he has certainly entirely destroyed it.

### Pituitary Extract.

Since the publication of Dr. Blair Bell's researches in the *British Medical Journal* a few months ago, the use of infundibular extract as a hypodermic injection has become widely popular. Whether it quite realises all that is hoped for, time has yet to show. Infundibular extract is administered in doses of about two grains of the fresh gland; and various firms are preparing sterilised solutions in glass capsules containing this or some similar dose. It is claimed that in conditions of shock this substance causes a rise of blood pressure even greater than that which it causes in the normal individual, and this effect lasts many hours. Accordingly, its use is advised in such states as post-operative shock, anæsthetic overdose, collapse from hæmorrhage, and so on. It also increases intestinal peristalsis, so that after severe abdominal operations when severe shock is a feature it combines the two advantages of preventing ileus and of raising blood-pressure. It also appears that this action on the intestinal muscle fibres, like that on the arterial coats, is more definitely obtained when atony and paresis are considerable. For this latter purpose another remedy that has lately been highly lauded is eserine salicylate. Of this drug 1-40 grain, with 1-60 grain of strychnine and 1-50 grain of digitalin, given hypodermically every hour, is said to act in a most satisfactory manner.

---

### Portable Urine Tests.

Dr. Seymour Taylor has recently drawn attention to a subject upon which comment was offered in these pages a short time ago (The Hospital, March 26, 1910, p. 739), namely, the importance of testing for albumin and sugar at the bedside in certain cases. His suggestions for overcoming the difficulties of portability and convenience differ from those of the American physician who was then quoted. Dr. Taylor strongly recommends the picric acid test for albumin introduced originally by Sir George Johnson. It can be applied without the use of a spirit lamp; it does not ruin the clothes or destroy leather and wood, as nitric acid does; and it is extremely accurate as well as delicate. An equal quantity of a saturated solution of picric acid is added to a small quantity of urine in a test tube; any turbidity indicates albumin. Furthermore, the same solution can be used for the detection of sugar; though for this purpose a spirit lamp or similar smokeless source of heat is required. After adding an equal quantity of the picric solution to a little urine, note whether albumin is precipitated. Then add a further similar quantity of caustic potash solution and agitate the mixture. Any albumin will be redissolved and a clear fluid of a brown sherry colour will result. Now if the upper portion of the mixture be boiled a deepening of the tint will take place, whether sugar is present or not; but if there be sugar, the colour will turn a very dark brown, almost black in fact. This tinge is quite unmistakable when it has once been seen. The test is not, says Dr. Taylor, the best test for sugar; but it is as good as some, and better than others.

